# Plasma complement component C2: a potential biomarker for predicting abdominal aortic aneurysm related complications

**DOI:** 10.1038/s41598-022-24698-1

**Published:** 2022-12-08

**Authors:** Tiam Feridooni, Abdelrahman Zamzam, Mariya Popkov, Muzammil H. Syed, Niousha Djahanpour, Mark Wheatcroft, Rawand Abdin, Mohammad Qadura

**Affiliations:** 1 Division of Vascular Surgery, St. Michael’s Hospital, Unity Health Toronto, 30 Bond Street, Suite 7-076, Toronto, ON M5B 1W8 Canada; 2 Department of Surgery, University of Toronto, Toronto, ON M5S 1A1 Canada; 3 Keenan Research Centre for Biomedical Science, Li Ka Shing Knowledge Institute, St. Michael’s Hospital, Unity Health Toronto, Toronto, ON M5B 1W8 Canada; 4 Department of Medicine, McMaster University, Hamilton, ON L8S 4K1 Canada

**Keywords:** Biomarkers, Aneurysm

## Abstract

Blood-based adjunctive measures that can reliably predict abdominal aortic aneurysm (AAA)-related complications hold promise for mitigating the AAA disease burden. In this pilot study, we sought to evaluate the prognostic performance of complement factors in predicting AAA-related clinical outcomes. We recruited consecutive AAA patients (n = 75) and non-AAA patients (n = 75) presenting to St. Michael’s Hospital. Plasma levels of complement proteins were assessed at baseline, as well as prospectively measured regularly over a period of 2 years. The primary outcome was the incidence of rapidly progressing AAA (i.e. aortic expansion), defined as change in AAA diameter by either 0.5 cm in 6 months, or 1 cm in 12 months. Secondary outcomes included incidence of major adverse aortic events (MAAE) and major adverse cardiovascular events (MACE). All study outcomes (AAA diameter, MACE and MAAE) were obtained during follow-up. Multivariable adjusted Cox regression analyses were performed to assess the prognostic value of plasma C2 levels in patients with AAA regarding rapid aortic expansion and MAAE and MACE. Event-free survival rates of both groups were also compared. Compared to non-AAA patients, patients with AAA demonstrated significantly higher plasma concentrations of C1q, C4, Factor B, Factor H and Factor D, and significantly lower plasma concentrations of C2, C3, and C4b (*p* = 0.001). After a median of 24 months from initial baseline measurements, C2 was determined as the strongest predictor of rapid aortic expansion (HR 0.10, *p* = 0.040), MAAE (HR 0.09, *p* = 0.001) and MACE (HR 0.14, *p* = 0.011). Based on the data from the survival analysis, higher levels of C2 at admission in patients with AAA predicted greater risk for rapid aortic expansion and MAAE (not MACE). Plasma C2 has the potential to be a biomarker for predicting rapid aortic expansion, MAAE, and the eventual need for an aortic intervention in AAA patients.

## Introduction

Abdominal aortic aneurysm (AAA) is a progressive cardiovascular disease with exceedingly high rates of morbidity and mortality, resulting in up to 200,000 annual deaths worldwide. Clinically, an AAA is defined as a 50% or greater increase in the diameter of the aorta^1^. Various risk factors have been linked with increased aortic wall degeneration, including but not limited to, old age, male sex, smoking, family history, hypertension, dyslipidemia, cardiovascular disease, and peripheral vascular disease^2^. Currently, the management of patients with known AAA includes serial surveillance (using either computed tomography (CT) or ultrasound) to monitor the maximum transverse diameter of the aneurysm^3^. Due to the life-threatening risk of rupture, repair of the AAA is generally indicated once the maximal transverse diameter reaches 5.0 cm in females and 5.5 cm in males^4,5^.

The progressive development of AAA is a dynamic process with a complex pathogenesis, but hallmarks include vascular smooth muscle cell apoptosis, oxidative stress, elastin fragmentation, extracellular matrix degradation, and inflammation^6,7^. Various circulating screening biomarkers have been proposed for the screening of AAA presence and size^2,8^; however, validated studies investigating biomarkers for prognostication of AAA-related complications have been scarce.

Previous studies have demonstrated the pivotal role played by the innate immune system in the progression of aortic aneurysms^9–11^. The complement system, which is part of the innate immunity and consists of more than thirty proteins and three pathways (the Classical, Lectin, and the Alternative)^12^, has elements that are found in every strata of the aneurysmal aortic tissue^13–15^. Since an association between plasma complement factors and aneurysm progression has previously been established^13,16^, we conducted this pilot study to: (1) investigate whether complement factors can serve as an adjunct in the prognosis of AAA-related complications and (2) study the potential of complement factors in facilitating the stratification of AAA patients as either high/low-risk for major adverse cardiac events (MACE), rapid aortic expansion, and/or major adverse aortic events (MAAE).

## Methods

### Patient recruitment and assessment

The first consecutive 75 patients encountered with asymptomatic infrarenal AAA and 75 controls without AAA presenting to ambulatory clinics at St. Michaels Hospital (Toronto, Canada) between May 2017 to May 2018 were included in this study. The patient's clinical data, physical exam, and abdominal aortic ultrasound were recorded upon initial encounter. Clinical data captured from patients included baseline demographics, history of cardiovascular diseases, cardiovascular risk factors (hypertension, hypercholesterolemia, and diabetes), and smoking status, as described previously^17^. The presence of AAA was verified through an ultrasound, with the diagnosis established by a vascular physician as per AAA-related clinical guidelines set forth by the Society of Vascular Surgery^3^. In short, patients were diagnosed with AAA if the observed aortic diameter on imaging was ≥ 3 cm. The control group consisted of patients presenting with non-AAA, vascular-related pathologies (varicose veins, thoracic outlet syndrome etc.) in addition to having an aortic diameter of < 3 cm.

### Patient selection criteria

Patients presenting with our study endpoints (see below), or one or more of the following indications were not eligible for inclusion: AAA diameter exceeding operative threshold (AAA diameter > 5 cm for females or > 5.5 cm for males), presenting with ruptured AAA, or presenting with symptomatic AAA (defined as symptoms that can be attributed to the aneurysm, such as abdominal pain or limb ischemia). Additionally, we excluded patients with a prior history of AAA repair, AAA secondary to mycotic or inflammatory etiology, sepsis (< 3 months) or malignancy. Lastly, patients with thoracic or thoracoabdominal aneurysms, as well as aortic dissections, were excluded.

### Ethics approval and blood sampling

Informed consent was obtained from all participants, and ethical approval was granted by St. Michael’s Hospital. Blood samples were obtained by venipuncture during the initial ambulatory visit. After adequate centrifugation, plasma samples were aliquoted and stored at − 80 °C. Levels of complement proteins (described below) were quantified using the same blood sample. All methods were carried out in accordance with relevant guidelines and regulations.

### Complement proteins and factors multiplex assay

Luminex MILLIPLEX MAP Kit Human Complement Magnetic Bead Panels 1 and 2 multiplex assay kits (EMD-Millipore; Billerica, MA, USA) were used to measure the plasma levels of the following proteins involved in the complement pathway: Complement C1q (C1q), Complement C2 (C2), Complement C3 (C3), Complement C4 (C4), Complement C4b (C4b), Complement C5 (C5), Adipsin Mannose-Binding Lectin (MBL), Complement Factor B (Factor B), Complement Factor D (Factor D), Complement Factor H (Factor H), and Complement Factor I (Factor I). The manufacturer's protocol was followed for the multiplex bead assays. Sample intra-assay Coefficients of Variability (CV) was < 10% while the inter-assay CV was 15%. Prior to any sample analysis, Fluidics Verification and Calibration bead kits (Luminex Corp) were used to calibrate the MagPix analyzer (Luminex Corp; Austin, Texas). At least 50 beads for each protein were acquired using Luminex xPonent software and analyzed using Milliplex Analyst software (v.5.1; EMD-Millipore).

### Measured outcomes

The primary outcome of this study was the rapid expansion of the AAA diameter observed during the follow-up period and defined as AAA size > 1 cm over 12 months or 0.5 cm over 6 months ^3^. Secondary outcomes included the incidence of major adverse aortic events (MAAE) and major adverse cardiovascular events (MACE). MAAE was defined as the composite incidence of elective AAA repair (open or endovascular repair), emergent AAA repair, AAA-related deaths, and AAA-induced complication (arterial thrombosis due to emboli from AAA, primary aorto-enteric fistula or primary aortocaval fistulas). MACE was defined as the composite incidence of cardiovascular-related mortality, stroke, or myocardial infarction.

### Two-year prospective follow-up

Over a period of 24 months after the initial baseline visit, patients were seen at 6-month or 12-month intervals (depending on their AAA size). This follow-up period was based on the AAA surveillance protocol recommended by the SVS guidelines^3^. During these follow-up visits, changes in clinical history or medications were recorded, AAA diameter was re-measured (via ultrasound), and the incidence of emergent AAA repair (secondary to the development of symptomatic AAA) or ruptured AAA were noted. Furthermore, the need for elective AAA repair (i.e. repair of AAA as per SVS guidelines—AAA diameter > 5 cm for females or > 5.5 cm for males) or repair of AAA due to rapid expansion were also recorded^3^.

### Statistical analysis

Baseline demographic and clinical characteristics were summarized as means and standard deviations (SDs) or numbers and proportions. Baseline differences between groups were calculated using independent t-test for continuous variables and chi-square test for categorical variables. Normality of plasma complement factor levels were assessed by the Kolmogorov–Smirnov test, and summarized as medians and interquartile ranges (IQRs) accordingly. Event rates for rapid AAA diameter expansion, MAAE, and MACE at 2 years were reported for the overall cohort and compared between AAA and non-AAA patient groups using chi-square test. Hazard ratios (HRs) and 95% confidence intervals (95% CIs) for events per one unit increase in plasma complement factors were calculated using univariable and multivariable models adjusted for age, sex, hypertension, dyslipidemia, smoking, and history of coronary artery disease. Receiver operator curve (ROC) analysis was conducted to identify a cut-off value for C2 that could facilitate stratification of AAA patients at-risk of adverse clinical outcomes into low versus high-risk groups. The cut-off value was chosen based on a high positive likelihood ratio (LR +) yielding a sensitivity above 90%. Overall event-free survival rates of both groups were displayed using Kaplan–Meier curves, and differences between curves were compared with a log-rank test. Significance was set at a two-tailed *p* < 0.05. All analyses were carried out using SPSS software version 23 (SPSS Inc., Chicago, Illinois, USA).

### Ethics statement

The studies involving human participants were reviewed and approved by Unity Health Toronto's Research Ethics Board. The patients/participants provided their written informed consent prior to participating in this study.

## Results

### Clinical characteristics

Baselines clinical characteristics of the recruited 75 AAA patients (50%) and 75 non-AAA patients (50%) are presented in Table 1. Overall, the mean age of the cohort was 67 (± 12) years. There were 101 (67%) male participants, 91 (61%) patients with hypertension, 94 (63%) with hypercholesterolemia, 23 (15%) with diabetes, 11 (7%) with renal insufficiency, 36 (24%) current smokers, 4 (3%) with history of congestive heart failure (CHF), 39 (26%) with history of coronary artery disease (CAD) and 13 (9%) with history of stroke. Patients with an AAA were significantly older than patients without an AAA (72 [± 8] vs. 61 [± 13], *p* = 0.001), had a higher percentage of active smokers (37% vs. 11%, *p* = 0.001), more likely to have hypercholesterolemia (80% vs. 47%, *p* = 0.001), renal insufficiency (13% vs. 1%, *p* = 0.004), CAD (39% vs. 13%, *p* = 0.01), and history of stroke (15% vs. 3%, *p* = 0.013). With regards to patient medical optimization, AAA patients were more likely to be on statins (81vs. 49%, *p* = 0.001), ACE inhibitors (62% vs. 31%, *p* = 0.001), and aspirin (60% vs. 28%, *p* = 0.001) when compared to non-AAA patients (Table 1).

**Table 1:** Clinical Characteristics of 150 Patients with and without AAA.

	Overall (n = 150)	AAA (n = 75)	No AAA (n = 75)	*p*-value
**Mean (SD)**				
Age	**67 (12)**	**72 (8)**	**61 (13)**	0.001*
**Frequency (%)** ‡				
Sex, Male	101 (67)	54 (72)	47 (63)	0.223
Hypertension	91 (61)	50 (68)	41 (55)	0.106
Hypercholesterolemia	**94 (63)**	**59 (80)**	**35 (47)**	0.001*
Diabetes	23 (15)	11 (15)	12 (16)	0.862
Renal Insufficiency	11 (7)	10 (13)	1 (1)	0.004
Current and Past Smokers	**36 (24)**	**28 (37)**	**8 (11)**	0.001*
History of congestive heart failure	4 (3)	2 (3)	2 (3)	1.000
History of coronary artery disease	**39 (26)**	**29 (39)**	**10 (13)**	0.001*
History of stoke	13 (9)	11 (15)	2 (3)	0.013
**Medication Frequency (%) ‡**				
ACEi/ARB	**66 (48)**	**46 (62)**	**20 (31)**	0.001*
ASA	**66 (44)**	**45 (60)**	**21 (28)**	0.001*
Beta blocker	35 (25)	23 (32)	12 (19)	0.075
CCB	23 (17)	15 (21)	8 (12)	0.168
HCTZ	9 (7)	5 (7)	4 (6)	0.654
Insulin	4 (3)	1 (1)	3 (5)	0.239
Oral Hypoglycemic	16 (12)	9 (13)	7 (11)	0.643
Statin	**92 (66)**	**60 (81)**	**32 (49)**	0.001*

Continuous variables are shown by mean (standard deviation). Frequencies and percentages were calculated for categorical variables; all numbers were rounded up with zero decimal place. *Represents significance difference between AAA and non-AAA groups, * p-value < *0.05*. All *p*-values were rounded to three decimal places. ‡ Differences between groups were compared using chi-square test. *AAA*, abdominal aortic aneurysm; *ACEi*, angiotensin-converting enzyme inhibitor; *ARB*, angiotensin receptor blocker; *ASA*, aspirin; *CCB*, calcium channel blocker; *HCTZ*, hydrochlorothiazide.

### Composition of circulating plasma complement factors in patients with and without and AAA

Compared to non-AAA patients, patients with an AAA had significantly higher median [IQR] levels of plasma factors C1q (59.5 ug/mL [32.8–67.4] vs. 41.1 ug/mL [30.6–57.9], *p* = 0.001), C4 (686 ug/mL [528–878] vs. 593 ug/mL [275–748], *p* = 0.001), Factor B (254 ug/mL [180–327] vs. 212 ug/mL [145–272], *p* = 0.001), Factor H (337 ug/mL [246–438] vs. 302 ug/mL [218–366], *p* = 0.002), and Factor D (3.59 ug/mL [2.14–5.68] vs. 2.24 ug/mL [1.32–3.34], *p* = 0.022). Conversely, compared to non-AAA patients, patients with AAA had a significantly lower median [IQR] levels of plasma factors C2 (0.26 ug/mL [0.19–0.41] vs. 0.33 ug/mL [0.28–0.42], *p* = 0.027), C3 (83.3 ug/mL [40.8–145] vs. 136 ug/mL [53–329], *p* = 0.014), and C4b (9.07 ug/mL [6.53–12.3] vs. 13.7 ug/mL [7.97–20.2], *p* = 0.006). No significant difference was noted in C5 (13.5 ug/mL [10.4–18.3] vs. 13.2 ug/mL [7.65–24.2], *p* = 0.666) and Factor I (25.9 ug/mL [16.6–52.8] vs. 22.4 ug/mL [14.7–41.3], *p* = 0.968) levels between both patient groups (Table 2).

**Table 2:** Plasma complement factors median (IQR) values. Units ug/ml.

	Overall (n = 150)	AAA (n = 75)	No AAA (n = 75)	*p*-value
C1q	48.4 (32.8–67.4)	59.5 (41.9–82.6)	41.1 (30.6–57.9)	0.001*
C2	0.31 (0.22–0.42)	0.26 (0.19–0.41)	0.33 (0.28–0.42)	0.027*
C3	110 (43–261)	83.3 (40.8–145)	136 (53.2–329)	0.014*
C4	646 (425–815)	686 (528–878)	593 (275–748)	0.001*
C4b	10.4 (6.95–17.1)	9.07 (6.53–12.3)	13.7 (7.97–20.2)	0.006*
C5	13.3 (8.24–22.3)	13.5 (10.4–18.3)	13.2 (7.65–24.2)	0.666
MBL	0.93 (0.14–2.33)	1.20 (0.17–2.89)	0.83 (0.10–1.90)	0.004*
Factor B	230 (165–292)	254 (180–327)	212 (145–272)	0.001*
Factor D	2.72 (1.82–4.21)	3.59 (2.14–5.68)	2.24 (1.32–3.34)	0.022*
Factor H	316 (223–408)	337 (246–438)	302 (218–366)	0.002*
Factor I	25.1 (15.2–46.5)	25.9 (16.6–52.8)	22.4 (14.7–41.3)	0.968

Continuous variables are shown by concentration in ug/mL (interquartile ranges). All numbers were rounded up with zero decimal place. *Represents significance difference between AAA and non-AAA groups, * p-value < *0.05*. All *p*-values were rounded to three decimal places. *AAA*, abdominal aortic aneurysm.

### Clinical outcomes

Complete, two-year follow-up data were available for 143 (95%) patients, with a mean duration of 22.0 (± 2.1) months. Over the follow-up period, 12 (8%) patients were observed to have rapid aortic expansion, 33 (22%) had a MAAE, and 30 (20%) had a MACE (Table 3).

**Table 3:** Distribution of adverse events in patients with AAA versus those without AAA during the 2-year follow-up.

	Overall (n = 150)	AAA (n = 75)	No AAA (n = 75)	*p*-value
Rapid expansion in AAA	**12 (8)**	**12 (16)**	**0 (0)**	0.001*
MAAE	**33 (22)**	**33 (44)**	**0 (0)**	0.001*
MACE	30 (20)	17 (23)	13 (17)	0.414

Continuous variables are shown in number (precent). * Represents significant difference between AAA and no AAA patients; *p* < 0.05; differences between groups were compared using chi-square test. All *p*-values were rounded to three decimal places. *AAA* abdominal aortic aneurysm; *MAAE* major adverse aortic event; *MACE* major adverse cardiac event.

### Association between complement proteins and study endpoints

**Figure 1:**
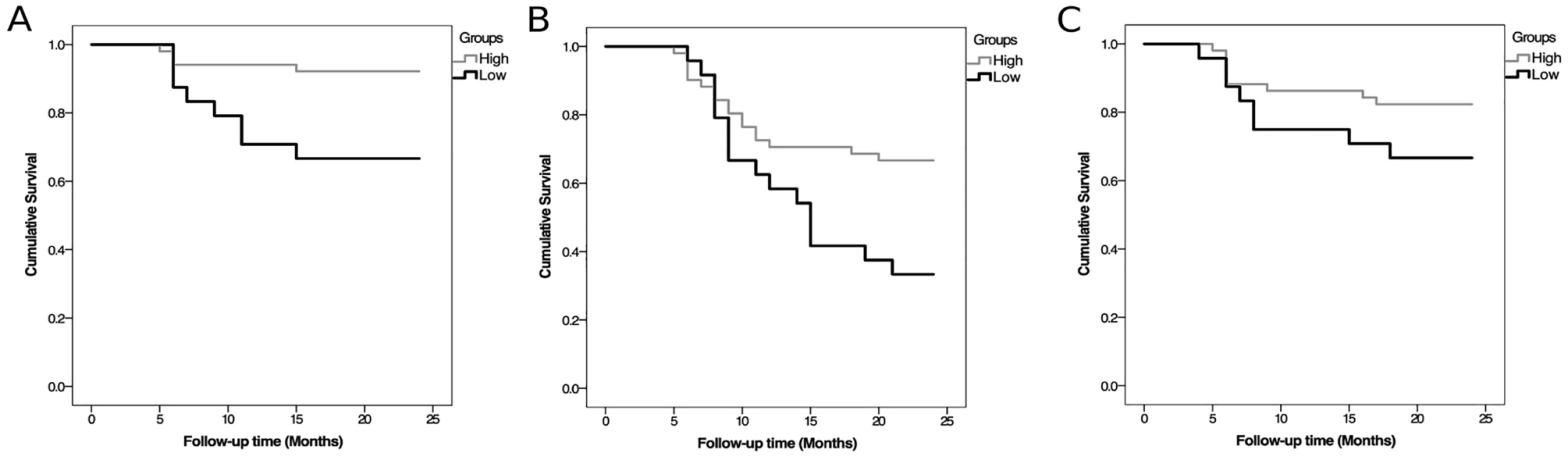
Cumulative event-free survival for in 75 patients with AAA divided into 2 groups, Low C2 group (n = 24) with C2 concentration < 0.202 ug/mL and High C2 group (n = 51) with C2 concentration > 0.202 ug/mL. (**A**) rapid AAA expansion (freedom from ΔAAA size > 1 cm/12 months or 0.5 cm/6 months) (**B**) MAAE and (**C**) MACE of all 75 patients diagnosed with AAA according to the levels of C2 levels (high versus low C2 levels), *p*-value = 0.001.

Among all the proteins investigated, C2 was singularly significantly predictive of all three clinical outcomes—rapid aortic expansion, MAAE and MACE. A decrease in plasma C2 (per ug/mL) was associated with significant increase in risk for rapid aortic expansion (adjusted HR 0.10 [95% CI 0.08–0.81], *p* = 0.040), MAAE (adjusted HR 0.09 [95% CI 0.03–0.26], *p* = 0.001) (Fig. 1B) and MACE (adjusted HR 0.14 [95% CI 0.03–0.63], *p* = 0.011) (Table 4). On the other hand, we noted that few of the investigated proteins were able to predict some but not all investigated outcomes. An increase in plasma Factor H (per ug/mL) was associated with an increase in the risk of MAAE (adjusted HR 1.12 [95% CI 1.05–6.70], *p* = 0.049) and risk of MACE (adjusted HR 0.51 [95% CI 0.30–0.87], p = 0.014) (Table 4). An increase in plasma C4b (per ug/mL) was associated with a decreased risk of MAAE (adjusted HR 0.35 [95% CI 0.16–0.76], *p* = 0.011) (Table 4). Lastly, an increase in plasma MBL (per ug/mL) was associated decrease in MACE (adjusted HR 0.54 [95% CI 0.30–0.87], *p* = 0.014) (Table 4). Since C2 was the only protein candidate that was significantly predictive of all primary and secondary study outcomes, it was selected for further analysis.

**Table 4:** Multivariable Cox proportional hazards model assessing the association between complement factors and rapid expansion in AAA (ΔAAA size > 1 cm/12 months or 0.5 cm/6 months), MAAE and MACE, adjusted for age, sex, smoking, and History of CAD, *p*-value < 0.05*. AAA*, abdominal aortic aneurysm; *MAAE***,** major adverse aortic event; *MACE* major adverse cardiac event; *HR* hazard ratio; *CI* confidence interval.

	Unadjusted			Adjusted		
	HR	95% CI	*P*-value	HR	95% CI	*P*
Rapid Aortic Expansion						
C1q	5.14	0.02–9.30	0.236	5.14	0.02–9.30	0.236
C3	1.07	0.41–2.81	0.885	1.07	0.41–2.81	0.885
C4	6.10	0.03–1.36	0.964	6.10	0.03–1.36	0.964
Factor B	0.98	0.06–15.2	0.992	0.98	0.06–15.2	0.992
Factor H	2.32	0.65–9.36	0.145	2.32	0.65–9.36	0.145
C2	*0.13*	*0.09–0.78*	*0.031*	*0.10*	*0.08–0.81*	0.040*
C4b	2.93	0.21–9.49	0.428	2.93	0.21–9.49	0.428
C5	*0.43*	*0.16–0.98*	*0.041*	0.41	0.19–1.32	0.097
Factor D	1.46	0.42–5.12	0.550	1.46	0.42–5.12	0.550
MBL	1.08	0.49–2.39	0.844	1.08	0.49–2.39	0.844
Factor I	1.06	0.31–3.61	0.921	1.06	0.31–3.61	0.921
**MAAE**						
C1q	3.97	0.84–7.13	0.147	3.95	0.83–7.12	0.147
C3	1.07	0.64–1.96	0.608	1.05	0.63–1.95	0.608
C4	1.86	0.45–7.63	0.391	1.84	0.42–7.62	0.391
Factor B	2.72	0.42–7.12	0.247	2.71	0.40–7.11	0.247
Factor H	*1.14*	*1.07–6.74*	*0.022*	*1.12*	*1.05–6.70*	0.049*
*C2*	*0.09*	*0.03–0.28*	*0.001*	*0.09*	*0.03–0.26*	0.001*
C4b	*0.36*	*0.17–0.79*	*0.011*	*0.35*	*0.16–0.76*	0.011*
C5	1.12	0.47–2.67	0.798	1.11	0.47–2.65	0.798
Factor D	1.35	0.53–3.52	0.533	1.34	0.52–3.51	0.533
MBL	1.79	0.95–3.37	0.069	1.78	0.92– 3.36	0.069
Factor I	0.91	0.41–2.02	0.814	0.91	0.41–2.01	0.814
**MACE**						
C1q	2.01	0.75–11.2	0.102	2.00	0.73–11.0	0.102
C3	0.63	0.35–1.15	0.134	0.62	0.32–1.14	0.134
C4	0.81	0.20–3.36	0.770	0.80	0.19– 3.35	0.770
Factor B	3.29	0.51–6.21	0.178	3.27	0.50–6.20	0.178
Factor H	3.71	0.48–8.25	0.502	3.71	0.46–8.24	0.502
C2	*0.15*	*0.04–0.60*	*0.008*	*0.14*	*0.03–0.63*	0.011*
C4b	*0.38*	*0.14–0.854*	*0.021*	0.36	0.12–1.02	0.057
C5	0.73	0.30–1.77	0.489	0.72	0.29–1.75	0.489
Factor D	1.18	0.43–3.27	0.784	1.17	0.42–3.26	0.784
MBL	*0.54*	*0.32–0.92*	*0.022*	*0.51*	*0.30–0.87*	0.014*
Factor I	0.72	0.29–1.73	0.461	0.70	0.28–1.72	0.461

### Correlation of plasma C2 levels and clinical variables

Among AAA and non-AAA patients, C2 plasma levels were not associated with any known established risk factor (age, sex, hypertension, hypercholesterolemia, diabetes, renal insufficiency, smoking, history of congestive heart failure, history of coronary artery disease, and history of stroke, *p*-value < 0.05) (Table 5). Similarly, C2 plasma levels were not associated with commonly used medications used to treat the listed risk factors (ACE inhibitors/ARBS, Aspirin, beta blockers, CCB, HCTZ, insulin, oral hypoglycemics, and statins, *p*-value < 0.05) (Table 5). However, median plasma C2 levels were significantly lower in patients with AAA (0.27 [0.21–0.45] vs. 0.39 [0.31–0.57], *p*-value = 0.002) relative to patients without AAA, (Table 5).

**Table 5:** Association between C2 levels and demographic data, cardiovascular risk factors and medications in 150 patients diagnosed with AAA and those without AAA.

	Mean (SD)	Correlation	*p*-value
Age	67 (12)	−0.151	0.073

	C2 levels (Median [IQR]) ‡		*p*-value
*AAA*	**AAA**	**No AAA**	
	**0.27 (0.21–0.45)**	**0.39 (0.31–0.57)**	0.002*
Sex	Male	Female	
	0.31 (0.24–0.41)	0.34 (0.23–0.44)	0.547
Hypertension	Yes	No	
	0.28 (0.22–0.39)	0.31 (0.20–0.45)	0.073
Hypercholesterolemia	Yes	No	
	0.35 (0.20–0.45)	0.27 (0.24–0.46)	0.062
Diabetes	Yes	No	
	0.31 (0.27–0.41)	0.31 (0.28–0.44)	0.414
Renal Insufficiency	Yes	No	
	0.34 (0.24–0.49)	0.35 (0.22–0.55)	0.649
Current and Past Smokers	Yes	No	
	0.33 (0.20–0.47)	0.29 (0.20–0.49)	0.389
History of congestive heart failure	Yes	No	
	0.32 (0.24–0.44)	0.20 (0.18–0.23)	0.516
History of coronary artery disease	Yes	No	
	0.34 (0.27–0.45)	0.31 (0.22–0.49)	0.211
History of Stroke	Yes	No	
	0.31 (0.24–0.44)	0.29 (0.18–0.46)	0.547
ACEi/ARB	Yes	No	
	0.32 (0.25–0.42)	0.33 (0.24–0.47)	0.470
Aspirin	Yes	No	
	0.31 (0.24–0.44)	0.33 (0.25–0.45)	0.774
Beta Blockers	Yes	No	
	0.33 (0.25–0.43)	0.31 (0.25–0.45)	0.767
CCB	Yes	No	
	0.32 (0.27–0.45)	0.31 (0.26–0.41)	0.549
HCTZ	Yes	No	
	0.31 (0.19–0.52)	0.32 (0.25–0.44)	0.955
Insulin	Yes	No	
	0.53 (0.32–0.66)	0.31 (0.25–0.43)	0.115
Oral hypoglycemic	Yes	No	
	0.30 (0.26–0.36)	0.33 (0.24–0.45)	0.508
Statin	Yes	No	
	0.30 (0.23–0.42)	0.34 (0.25–0.45)	0.087

Continuous variables are shown in mean (standard deviation). ‡Data summarized as medians and interquartile ranges (IQRs). *Represents significant difference between AAA and no AAA patients; *p* < 0.05; differences between groups were compared using chi-square test. All *p*-values were rounded to three decimal places. *AAA*, abdominal aortic aneurysm.

### Prognostication of study outcomes based on C2 levels at presentation

Based on the ROC curve, we identified a C2 concentration of 0.202 ug/mL (AUC of 0.709 (*p* = 0.010, 95% CI 0.641–0.763), likelihood ratio (LR) + 5.34, 91% sensitive, and 62% specific) as the optimal cutoff value to facilitate stratification of AAA patients at-risk of clinical complications into low-risk vs high-risk groups. Utilizing this cutoff value, AAA patients (n = 75) were divided into 2 groups, (1) Low C2 group (n = 24) with C2 concentration < 0.202 ug/mL and (2) High C2 group (n = 51) with C2 concentration > 0.202 ug/mL. The clinical characteristics of both these groups are highlighted in Table 6.

**Table 6:** Comparison of the Clinical Characteristics for AAA Patients with high and low plasma levels of C2.

	Low C2 (n = 24)	High C2 (n = 51)	*p*-value
**Mean (SD)**			
Age	73 (8)	72 (7)	0.949
**Frequency (%)**‡			
Sex, Male	19 (79)	35 (69)	0.343
Hypertension	13 (57)	37 (73)	0.173
Hypercholesterolemia	20 (87)	39 (77)	0.299
Diabetes	4 (17)	7 (14)	0.737
Renal Insufficiency	2 (8)	8 (16)	0.382
Current and Past Smokers	10 (42)	18 (36)	0.595
History of congestive heart failure	1 (4)	1 (2)	0.580
History of coronary artery disease	6 (25)	23 (45)	0.095
History of Stroke	3 (13)	8 (16)	0.716
**Event rate n (%) **^**α**^			
Rapid expansion in AAA	**8 (33)**	**4 (8)**	0.005*
MAAE	**16 (67)**	**17 (33)**	0.007*
MACE	8 (33)	9 (18)	0.130

Continuous variables are shown by mean (standard deviation). ‡ Frequencies and percentages were calculated for categorical variables; ^**α**^ Event rate variables are shown in number (precent). *Represents significant difference between Low C2 and High C2 patients; *p* < 0.05; differences between groups were compared using chi-square test. All *p*-values were rounded to three decimal places. *AAA* abdominal aortic aneurysm; *MAAE* major adverse aortic event; *MACE* major adverse cardiac event.

Among AAA patients, a higher rate of rapid aortic expansion (33% vs. 4%, p = 0.001) and MAAE (67% vs. 33%, *p* = 0.007) was noted in patients with low C2 levels compared to the High C2 group. No significant difference was noted in the incidence of MACE (33% vs. 18%, *p* = 0.130) among AAA patients with high versus low C2 levels.

Kaplan–Meier analysis demonstrated that low plasma levels of C2 (< 0.202 ug/mL) can reliably stratify patients into those most likely to undergo rapid aortic expansion (*p* = 0.005; log-rank = 7.78) (Fig. 1A) as well as MAAE (*p* = 0.014; log-rank = 6.02) (Fig. 1B), but not MACE (*p* = 0.132; log-rank = 2.26) (Fig. 1C). Freedom from rapid expansion in AAA at 1 and 2 years were 94% and 92% in the high C2 group, respectively, and 71% and 67% in the low C2 group, respectively (Fig. 1A). MAAE-free survival rates at 1 and 2 years were 71% and 67% in the high C2 group, respectively, and 58% and 33% in the low C2 group, respectively (Fig. 1B). Finally, MACE-free survival rates at 1 and 2 years were 86% and 82% in the high C2 group, respectively, and 75% and 67% in the low C2 group, respectively (Fig. 1C).

## Discussion

**Figure 2:**
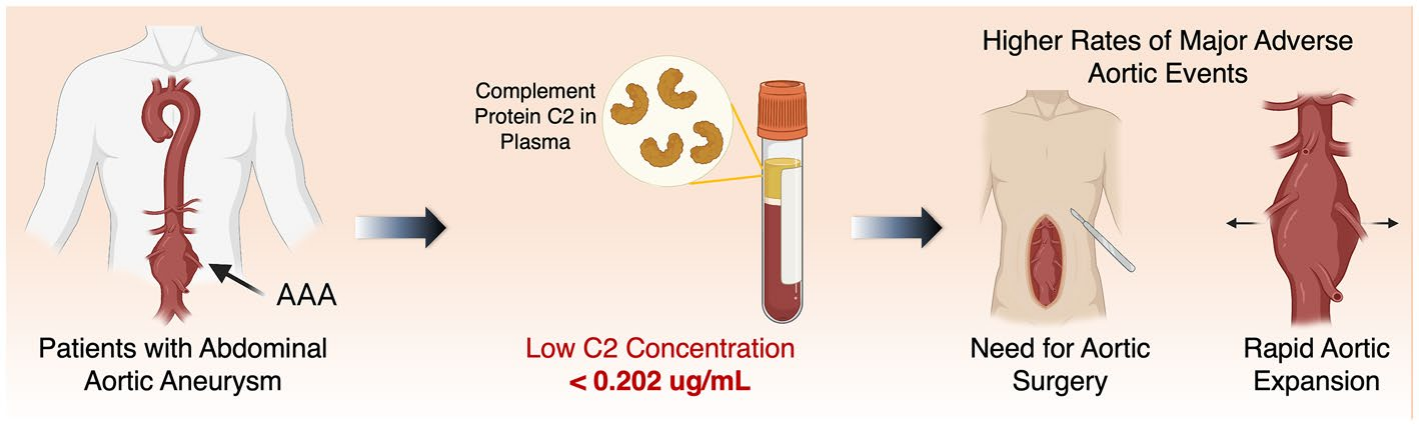
The clinical workflow for the use of complement factor 2 (C2) as a prognostic biomarker for major adverse aortic events (MAAE) and rapid aortic expansion in patients with abdominal aortic aneurysm (AAA). Created using BioRender with permission.

In this study, we demonstrated significant differences in plasma levels of component factors in AAA patients versus non-AAA patients. Our analysis demonstrated that baseline C2 was a reliable predictor of all three measured outcomes in this study, including rapid aortic expansion, MAAE and MACE over a two-year follow-up period. Based on Kaplan–Meier analysis data, measuring C2 levels at baseline may aid and serve as a potential biomarker for stratifying patients at risk of rapid aortic expansion or MAAE (Fig. 2).

As various elements of the complement system are found in different strata of the aortic tissue, a growing body of evidence demonstrates the active involvement of the complement system in acute cardiovascular events and aortic disease^13–15^. In murine animals, complement C3a and C5a depletion were protective against AAA formation^13^. In contrast, Zagrapan et al. found significantly increased levels of circulating C5a factor in the plasma levels of AAA patients compared to healthy patients^16^. Furthermore, Zagrapan et al. also linked plasma C5a levels with aneurysm progression, thereby conferring a potential role for complement factors as an adjunct for the prognosis of patients with AAA^16^. In this study, we demonstrated a significant difference in complement factors in the Classical pathway (C1q, C2, C4 and C4b), Lectin pathway (MBL) and Alternative pathway (C3, Factor B and Factor D) among patients with and without an AAA.

Additionally, we demonstrated an association between low circulating C2 levels and increased risk for rapid aortic expansion and MAAE in patients with AAA. In comparison, Hinterseher et al. demonstrated an increase in gene expression of *C1Qa*, *C1Q* and *C2* and a decrease in expression of C2 inhibitor *SERPING1* in human aortic aneurysmal tissue^18^. Furthermore, they noted an increase in complement protein C2 staining in cells of aortic aneurysmal tissue^18^.

The pathogenesis behind our findings (i.e. the association between low levels of C2 in plasma and an increased expression of C2) within aneurysmal aortic tissue still needs to be investigated further. Previously, the Classical pathway has been shown to be independently activated by pentraxins, such as C-reactive protein (CRP), which has also been linked to aortic expansion in patients with AAA^19,20^. Homozygous C2 deficiency, in addition to its association with severe infections and rheumatic disease, has also been linked with various forms of vasculitis with cutaneous and gastrointestinal manifestations^21,22^. However, a direct link between C2 deficiency and aneurysm formation is yet to be established. Thus, the biological role of C2 in aortic aneurysm progression would undoubtedly be an area of interest that would warrant further investigation.

To date, numerous circulating biomarkers have been investigated as potential predictive factors for AAA expansion and rupture. These markers can be categorized into those involved in the coagulation pathway^23–30^, extracellular matrix turnover and matrix degrading enzymes^23,30–42^ and lipids^25,43–46^. Furthermore, there have been various circulating biomarkers involved in the immune response system that have been investigated for an association with AAA expansion and/or rupture, which include, CRP^24,25,35,46–49^, interleukin-1β^50^, interleukin-2^50^, interleukin-6^46,50,51^, interleukin-8^50^, interferon-gamma^52^, leukocytes^24^, macrophage inhibiting factor^23,53^, neutrophil gelatinase-associated lipocalin^54^, osteopontin^55^, osteoprotegerin^56^, peroxiredoxin^57^, tumour necrosis factor-α^46,50^, tumour necrosis factor-like weak inducer of apoptosis^58^ and C5a^16^. The lack of data on the role of the complement system in aortic expansion led to further analysis of the relationship between circulating complement factors and aortic expansion.

The clinical decision-making for AAA treatment can be complicated by intrapatient and interpatient variations^59^. Furthermore, recent studies have cast doubt over whether the maximum diameter alone should guide the treatment of patients with AAA^60^. Notably, circulating biomarkers have also been greatly emphasized due to their capacity to provide important prognostic information about subsequent aortic behaviour, thereby allowing for more patient-specific management^8,48,61–64^. At the time of writing this paper, more accurate prognostic predictors are needed to guide stratifying patients into those at risk for rupture rather than relying on diameter alone, as some small AAA are known to rupture, while some large AAAs can remain dormant for some time^4,65^. The current SVS guidelines suggest surveillance imaging for AAAs measuring 3.0–3.9 cm, 4.0–4.9 cm and 5.0–5.4 cm at 3-year, 12-month and 6-month intervals, respectively^3^. In contrast, our findings indicate that there may be a subgroup of AAA patients (those with low circulating plasma C2 at higher risk of rapid aortic expansion and MAAE) who may benefit from careful oversight and more frequent follow-up. Furthermore, circulating C2 levels may be utilized as a part of the clinical decision-making process to help reduce the risks associated with AAA treatment, particularly in high-operative-risk patients, until the risk of rupture is believed to outweigh the operative risk^4^. While our findings regarding plasma C2 levels may add to the potential biomarkers that can be used to prognosticate patients with AAA, further validation in a larger and more heterogeneous patient cohort is still required.

Limitations include the single-center nature of our study and the unaccounted study outcomes in patients lost to follow-up. A larger and more diverse sample size with prolonged follow-up may prove insightful in evaluating the true prognostication potential of C2 in patients with AAA, as this was a pilot study to determine whether the role of complement factors in AAA disease warrants further investigation. Future studies investigating the biological role of C2 in aneurysmal aortic tissue are also warranted. Not all complement factors and their associated activated forms and substrates were investigated in this trial. Lastly, there may have additional confounding factors aside from the ones measured in this study that may correlate with plasma C2 levels, which will surely need to be examined in future studies.

In conclusion, we demonstrated that C2 has a strong predictive potential for AAA-related complications despite adjusting for confounding factors. Provided our findings are validated, circulating plasma C2 may be used in the future as a viable adjunct blood-based biomarker for the identification of AAA patients at high risk of rapid expansion and MAAE.

## Data Availability

All data generated or analyzed during this study are included in this published article.
